# Visualizing Neuropharmacological Effects of Guanfacine Extended Release in Attention Deficit Hyperactivity Disorder Using Functional Near-Infrared Spectroscopy

**DOI:** 10.3389/fnrgo.2021.657657

**Published:** 2021-07-07

**Authors:** Takahiro Ikeda, Akari Inoue, Daisuke Tanaka, Tamao Hashimoto, Stephanie Sutoko, Tatsuya Tokuda, Yasushi Kyutoku, Atsushi Maki, Takanori Yamagata, Ippeita Dan, Yukifumi Monden

**Affiliations:** ^1^Department of Pediatrics, Jichi Medical University, Shimotsuke, Japan; ^2^Applied Cognitive Neuroscience Laboratory, Faculty of Science and Engineering, Chuo University, Bunkyo, Japan; ^3^Center for Exploratory Research, Research and Development Group, Hitachi, Ltd., Hiki, Japan; ^4^Research and Development Initiatives, Applied Cognitive Neuroscience Laboratory, Chuo University, Bunkyo, Japan; ^5^Center for Development of Advanced Medical Technology, Jichi Medical University, Shimotsuke, Japan

**Keywords:** attention deficit hyperactivity disorder, cortical hemodynamics, developmental disorder, dorsolateral prefrontal cortex, optical topography, angular gyrus

## Abstract

**Objective:** In the current study, we explored the neural substrate for acute effects of guanfacine extended release (GXR) on inhibitory control in school-aged children with attention deficit hyperactivity disorder (ADHD), using functional near-infrared spectroscopy (fNIRS).

**Methods:** Following a GXR washout period, 12 AD HD children (6–10 years old) performed a go/no-go task before and 3 h after GXR or placebo administration, in a randomized, double-blind, placebo-controlled, crossover design study. In the primary analysis, fNIRS was used to monitor the right prefrontal cortical hemodynamics of the participants, where our former studies showed consistent dysfunction and osmotic release oral system-methylphenidate (OROS-MPH) and atomoxetine hydrochloride (ATX) elicited recovery. We examined the inter-medication contrast, comparing the effect of GXR against the placebo. In the exploratory analysis, we explored neural responses in regions other than the right prefrontal cortex (PFC).

**Results:** In the primary analysis, we observed no significant main effects or interactions of medication type and age in month (two-way mixed ANCOVA, *Fs* < 0.20, all *ps* > .05). However, in the *post-hoc* analysis, we observed significant change in the oxy-Hb signal in the right angular gyrus (AG) for inter-medication (one sample *t*-test, *p* < 0.05, uncorrected, Cohen's *d* = 0.71).

**Conclusions:** These results are different from the neuropharmacological effects of OROS-MPH and ATX, which, in an upregulated manner, reduced right PFC function in ADHD children during inhibitory tasks. This analysis, while limited by its secondary nature, suggested that the improved cognitive performance was associated with activation in the right AG, which might serve as a biological marker to monitor the effect of GXR in the ADHD children.

## Introduction

Attention deficit hyperactivity disorder (ADHD) is one of the most common childhood behavioral disorders. Its global community prevalence is from 2 to 9% of the child population (Sayal et al., 2018). ADHD is related to deficits in overall executive functions, especially in response inhibition and attention (Barkley, 1997). ADHD symptoms appear during the preschool years and often persist into adulthood. Thus, early diagnosis and intervention are important for long-term prognoses.

The recommended treatments for ADHD children comprise behavioral therapy and pharmacotherapy. In Japan, osmotic release oral system-methylphenidate (OROS-MPH), atomoxetine hydrochloride (ATX), lisdexamfetamine dimesylate (LDX), or guanfacine extended release (GXR) can be administered to children with ADHD as a monotherapy. These drugs improve core symptoms of ADHD (Frampton, 2018). Drugs other than GXR mainly inhibit the reuptake of monoamine (Easton et al., 2007). In previous studies, they have been said to upregulate hypofunction in the monoamine systems, including mesocortical dopamine pathways from the ventral tegmental area to the prefrontal cortex (PFC), mesolimbic dopamine pathways from the ventral tegmental area to several limbic structures, with the largest projection to the nucleus accumbens, and noradrenaline pathways from the locus coeruleus with axonal projections to the prefrontal and parietal cortices (Rubia et al., 2011).

Neuroimaging studies, using functional magnetic resonance imaging (fMRI*)*, have examined pharmacological effects in ADHD from the perspective of brain function. These studies have revealed that administration of ADHD therapeutic agents is related to the improvement of neural response in the PFC, which is one of the neural bases of inhibition functions as a core deficit in ADHD (Rubia et al., 2011). Our previous studies, using functional near-infrared spectroscopy (fNIRS*)* on young children with ADHD, have also shown that the right prefrontal hypoactivation was related to the dysfunction of response inhibition in ADHD children and normalization of the hemodynamic responses in the right middle and inferior gyri with OROS-MPH and ATX medication during the inhibition task (go/no-go task) (Monden et al., 2012a,b; Nagashima et al., 2014). On the other hand, GXR, a selective α_2A_-adrenoreceptor agonist, improves core deficits in ADHD, as do other ADHD drugs (van Stralen, 2020) but might have a different pharmacological mechanism from other ADHD drugs. α_2A_-adrenoreceptor agonists, such as clonidine, stimulate the presynaptic α_2A_-receptors to couple *via* G-protein to several effectors, including the inhibition of adenylate cyclase, and restrict the release of noradrenaline in the central nervous system (Aitkenhead et al., 2007). However, the precise mechanism of GXR action in the treatment of ADHD is still not fully understood.

Previous neuroimaging studies have shown that GXR activates the frontal cortex in animals (Avery et al., 2000) and in healthy young adult persons (Clerkin et al., 2009; Schulz et al., 2013). To our knowledge, only one fMRI study has revealed pharmacological neuromodulation by GXR on inhibition function in human ADHD subjects (Bédard et al., 2015) but provided negative evidence for activation in the PFC. Thus, the present study aims to reveal the neuropharmacological effects of GXR monotherapy on ADHD children, using fNIRS in a randomized, double-blind, crossover, placebo-controlled design. First, in the primary analysis, we focused on the right PFC, which, in ADHD children, was acutely normalized after administration of OROS-MPH and ATX in our previous reports. Second, in the exploratory analysis, we explored neural responses in other lateral cortical areas.

## Methods

### Participants and Ethics

We recruited clinically referred, right-handed Japanese children aged 6–10 years, who were diagnosed as ADHD based on the DSM-5 criteria from Jichi Medical University (Shimotsuke, Tochigi, Japan), the International University of Health and Welfare (Nasushiobara, Tochigi, Japan), and Rehabilitation Center, International University of Health and Welfare (Otawara Tochigi, Japan) for the study. Consequently, 12 ADHD children became the subjects of the study (Table 1). The subjects were on oral GXR (1 mg/day) for at least 8 weeks at the time of consent and had body weights of 17–38 kg at the time of consent (Table 1). Their intelligence quotient (IQ) scores were estimated based on the Wechsler Intelligence Scale for Children Fourth (WISC-IV), and the full-scale IQ (FSIQ) of all subjects exceeded 70 in this study (Table 1). Exclusion criteria were any treatment with ADHD medication other than GXR.

**Table 1:** Demographics and clinical profiles of subjects.

	**Age (years)**	**Gender male: female**	**Duration of GXR exposure (months)**	**Body weight (kg)**	**WISC-IV FSIQ**
*M*	8.2	11:1	16.4	24.4	100.3
*SD*	1.5	–	6.6	5.1	11.9
range	6–10	–	8–24	17.6–35.4	83–122

*M, mean; SD, standard deviation*.

All the subjects and their parents gave oral consent to participation in the study, and written consent was obtained from the parents. Subjects had the right to opt out of the study. The study was approved by the Ethics Committees of Jichi Medical University Hospital and the International University of Health and Welfare (CRB3180003), and complies with the latest version of the Declaration of Helsinki. Data were anonymized, and no patient identifying information was included. The study was registered to the specified clinical trials (clinical trial plan number: jRCTs 031190060) as “Optical Topography-based Neuropharmacological effect of Guanfacine Hydrochloride in ADHD Children.”

### Experimental Procedure

We tested the effects of GXR in a randomized, double-blind, crossover, placebo-controlled design study while the subjects performed a go/no-go task. Twelve ADHD subjects were examined two times (the times of days for both measurements were scheduled to be as close as possible) at either of the two hospitals, at least 4 days apart, but within 30 days.

On each examination day, the subjects performed two sessions: one before medication (SPD503 1 mg GXR or placebo) administration, and the other at 3 h after medication. The subjects who were administered GXR on the 1st day were administered a placebo on the 2nd day, whereas those administered a placebo on the 1st day were administered GXR on the 2nd day. We used a randomized order to avoid order effects, and the order was counterbalanced across all the subjects. A crossover design was employed to reduce the required sample size and influence of interindividual variations, and a double-blind design was employed to avoid the influence of bias from expectations of the subjects, investigators, etc.

After a washout period of 4 days, the subjects each underwent a pre-administration session. To minimize the carryover effect, we set the washout period to 4 days because it should be at least five times the half-life of the drug: Half-life of GXR was reported as 14–17 h (Boellner et al., 2007).

### Experimental Design

**Figure 1:**
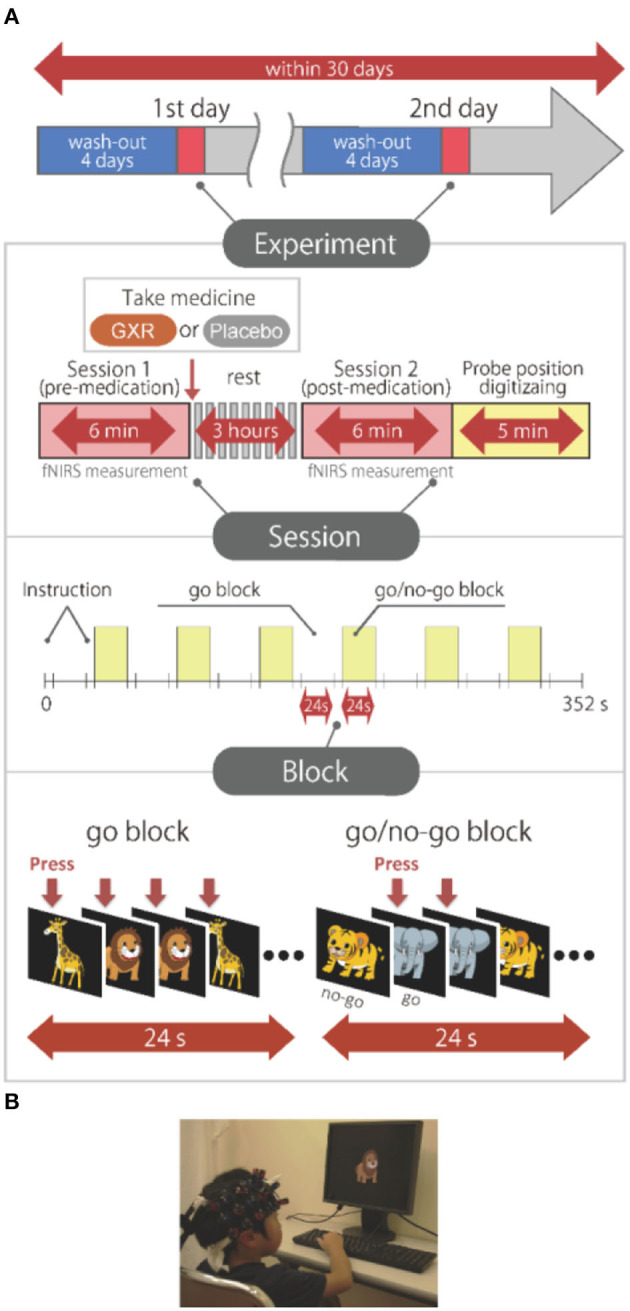
Experimental design. **(A)** Summary of experimental design. All the subjects were examined on 2 days. A schematic showing the flow of pre- and post-medication administration sessions for the subjects. Brain activity was measured with functional near-infrared spectroscopy (fNIRS) measurements during the go/no-go task. **(B)** fNIRS measurements. Brain activity was measured while ADHD and control subjects performed the go/no-go task. We have obtained parental permission to publish the image of the participation in the figure.

We measured cortical activation with fNIRS during a go/no-go task. The experimental design was the same as the design in Monden et al. (2012a). Specifically, we selected the block-design go/no-go task used in the previous studies (Monden et al., 2012a,b; Nagashima et al., 2014) (Figure 1). Four different animal pictures were displayed for the subject on a desktop computer screen. The go/no-go task consisted of six block sets, each containing alternating go (baseline) and go/no-go (target) blocks. In the go block, the subjects were asked to press the button when they saw either of two animal pictures randomly displayed, as instructed, with the sentence “when you see each picture, you should press the space key as quickly as you can.” In the go/no-go block, two other animal pictures were displayed. However, the subjects were asked to press the button when they saw specific animal pictures (go trial) and asked to not press the button for the other animal picture (no-go trial), as instructed with the sentence “when you see the no-go picture, you should not press the space key.” Both of the blocks lasted 24 s after the instructions were displayed for 3 s, resulting in an overall block-set time of 54 s and a total session time of 6 min. The pictures were displayed sequentially for 800 ms with an interstimulus interval of 200 ms in both go and go/no-go blocks. To ensure their understanding of the instructions, each subject performed a practice block before the measurements. E-Prime 2.0 (Psychology Software Tools) was used to generate the stimuli and collect the responses.

### fNIRS Measurement

We used a multichannel fNIRS system, ETG-4000 (Hitachi Corporation, Kashiwa, Japan), which employs two wavelengths of near-infrared light (695 and 830 nm) to measure hemodynamics representing cortical activation. Optical data were analyzed based on the modified Beer-Lambert law (Maki et al., 1995). This method enabled us to calculate signals, reflecting the oxygenated hemoglobin (oxy-Hb) and deoxygenated hemoglobin (deoxy-Hb) signal changes, calculated in units of millimolar·millimeter (mM·mm) (Maki et al., 1995). The sampling rate was set at 10 Hz. We analyzed the oxy-Hb signal as in Monden et al. (2012b).

### fNIRS Probe Placement

**Figure 2:**
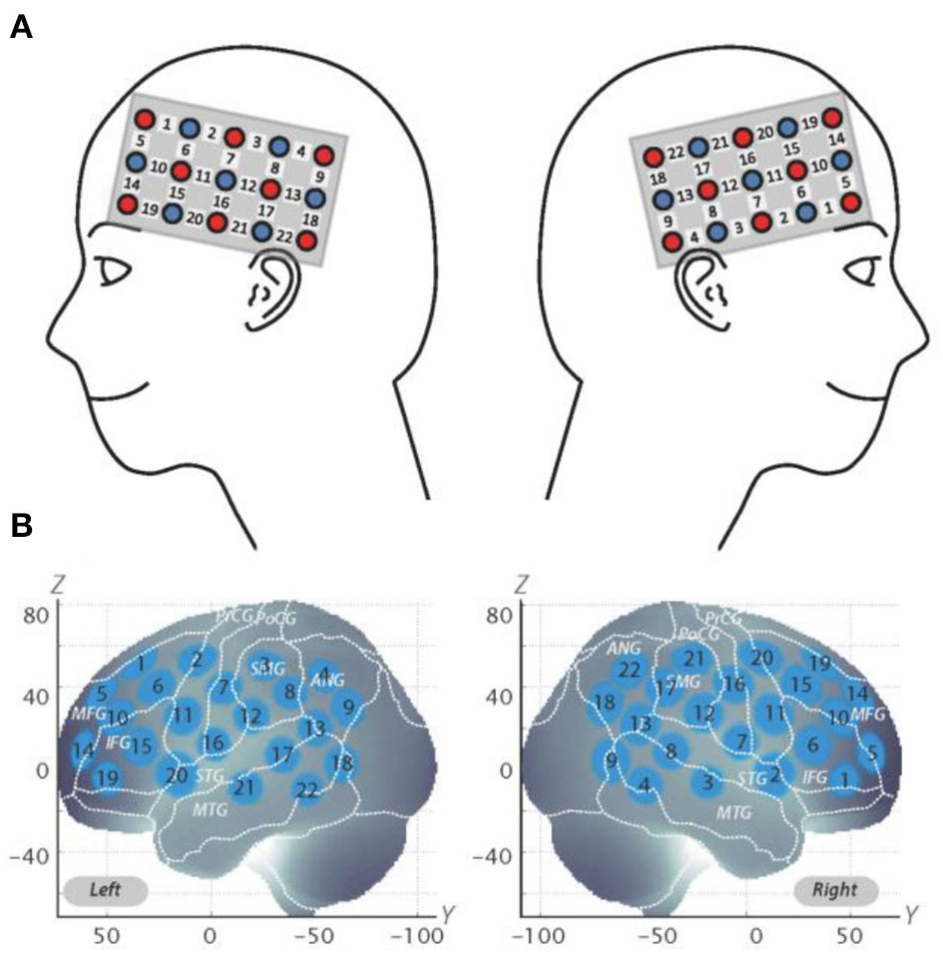
Spatial profiles of fNIRS channels. **(A)** Left- and right-side views of the probe arrangements are exhibited with fNIRS channel orientation. Detectors are indicated with blue circles, illuminators with red circles, and channels with white squares. Corresponding channel numbers are shown in black. **(B)** Channel locations on the brain are exhibited for both left- and right-side views. Probabilistically estimated fNIRS channel locations (centers of blue circles) for subjects and their spatial variability (standard deviation: radii of the blue circles) associated with the estimation are depicted in Montreal Neurological Institute (MNI) space.

The fNIRS probes were set to cover the lateral prefrontal cortices and parts of the frontal, parietal, and temporal lobes in reference to previous studies (Monden et al., 2012a,b; Nagashima et al., 2014), resulting in 22 channels (CH) per hemisphere (Figure 2A). Specifically, we used two sets of 3 ×5 multichannel probe holders that consisted of eight illuminating and seven detecting probes arranged alternately at an inter-probe distance of 3 cm. The midpoint of a pair of illuminating and detecting probes was defined as a channel location. We placed the bilateral probe holders in the following manner: (1) their upper anterior corners, where the left and right probe holders were connected by a belt, were symmetrically placed across the sagittal midline; (2) the lower anterior corners of the probe holder were placed over the supraorbital prominence; and (3) the lower edges of the probe holders were attached at the upper part of the auricles (Figure 2A). For spatial profiling of fNIRS data, we adopted virtual registration (Tsuzuki et al., 2012) for registering fNIRS data to Montreal Neurological Institute (MNI) standard brain space (Brett et al., 2002). Specifically, the positions for channels and reference points, which included the Nz (nasion), Cz (midline central), and left and right preauricular points, were measured, using a three-dimensional digitizer in real-world (RW) space. We affine-transformed each RW reference point to the corresponding MRI-database reference point and then replaced it with MNI space. Adopting the same transformation parameters enabled us to obtain the MNI coordinate values for the fNIRS channels in order to obtain the most likely estimate of the location of given channels for the group of the participants and the spatial variability associated with the estimation (Figure 2B). Then, the estimated locations were anatomically labeled, using a MATLAB® function that reads anatomical labeling information coded in a microanatomical brain atlas [LBPA40 and Brodmann] (Tsuzuki and Dan, 2014).

### Preprocessing of fNIRS Data

The individual timeline data for the oxy-Hb and deoxy-Hb signals of each channel were preprocessed with a first-degree polynomial fitting, a 0.01-Hz high-pass filter to exclude baseline drift and a 0.8-Hz low-pass filter to exclude heartbeat pulsations. From the preprocessed time series data, we computed channel-wise and subject-wise contrasts by calculating the intertrial mean of differences between the oxy-Hb signals for target periods (4–24 s after the go/no-go block onset) and baseline periods (14–24 s after the go-block onset).

### fNIRS Data Analysis 1: Region-of-Interest Analysis

We previously reported that right inferior frontal gyrus (IFG)/middle frontal gyrus (MFG) activation in ADHD children during a go/no-go task was acutely normalized after administration of OROS-MPH or ATX (Monden et al., 2012a,b; Nagashima et al., 2014). Accordingly, we set the right CH10, located at the right IFG/MFG, as a region of interest (ROI) for this analysis. We analyzed oxy-Hb signals at the ROI as a primary analysis.

For the six go/no-go blocks, two raters (AI and TH) manually inspected the time-series data and removed the blocks with sudden, obvious, discontinuous noise generated by the motions of the subjects. We also excluded data for the subjects with more than three out of six blocks removed.

We generated intra-medication contrasts, which is the difference between post- and pre-medication contrasts, for each medication (i.e., placebo^post−pre^ and GXR^post−pre^ contrasts). Using the contrast, we performed a two-way analysis of covariance (ANCOVA) for the primary analysis on the intra-medication of each medication with within-subject effect for a medication type (GXR vs. placebo), between-subject effect for medication order (GXR-to-placebo vs. placebo to GXR), and age in months as a time invariant covariate. A previous fMRI study of children reported that brain activity in the right prefrontal cortex during the execution of a cognitive control task correlates with age in months (Durston et al., 2006). Considering this previous study, age in months was used as a covariate. As for within-subject effects, interaction between the independent variables (IV) and a covariate was used for adjustment as described by Tabachnick and Fidell (2007). Therefore, interaction term(s) among the IVs and the covariate were included in the general linear model (GLM). Regarding within-subject effects (the medication type and interaction between the IVs) in the current study, an interaction term corresponding to each effect (medication type^*^age and medication type^*^medication order^*^age, respectively) was specifically used for adjustments but not to interpret the effect of covariate. The statistical threshold was set at *p* < 0.05.

### fNIRS Data Analysis 2: Exploratory Analysis

Furthermore, we analyzed the oxy-Hb signals of all channels in a channel-wise manner as an exploratory analysis. Oxy-Hb signals of all the channels were corrected for motion artifacts, using the correlation-based signal improvement (CBSI) method (Cui et al., 2010). We generated the same contrast as described in the previous section.

One-sample *t*-tests (two tails) against 0 (equivalent to paired *t*-tests) were performed on the contrast between GXR^post−pre^ and placebo^post−pre^ with an alpha level set at 0.05 for the exploratory analysis. We selected the one-sample *t*-test because there were only negligible effects of medication order and age in the primary analysis. We measured multiple channels (44 channels) in this study. In the exploratory analysis, which analyzed the oxy-Hb signals of all the channels in a channel-wise manner, the number of hypotheses increased up to the number of channels. Such multiple comparisons entail an increased risk of Type I errors called “family-wise errors.” Therefore, the effective multiplicity (M_eff_*)* method was used for family-wise error correction (Uga et al., 2015).

### Behavioral Data Analysis

We assessed the behavior data based on the following parameters: (a) reaction time (RT) for go trials; (b) accuracy (ACC) for go trials (omission error); and (c) ACC for no-go trials (commission error). In the task design of this study, the participants were not supposed to press the button in the no-go trials. Accordingly, there was no reaction time in the no-go trials. Thus, we calculated the mean RT for each participant, using the average RTs for correct go trials in the go/no-go block. We computed accuracy as a ratio for go trials by dividing the number of correct responses (i.e., the subjects pressed the button in go trials) by the total number of the go trials in the go/no-go block. Similarly, we computed accuracy for no-go trials by dividing the number of correct inhibitions (i.e., subjects did not press the button in no-go trials) by the total number of no-go trials in the go/no-go block.

We averaged ACCs and RTs across go/no-go blocks and generated the same contrast as described for fNIRS data in the previous section. We performed a one-sample *t*-test (two tails) against 0 on this contrast with an alpha level set at 0.05 for the second analysis. For all statistical analyses, we used the SPSS statistics (version 25 for Windows; SPSS Inc., Chicago, IL) software package.

## Results

### fNIRS Data 1: ROI Analysis

We set the right CH10 as an ROI for this analysis; this channel was located at the border region between the right MFG and IFG (MNI coordinates: *x, y, z* (*SD*): 48, 44, 25 (12), MFG, 57%; IFG 43%; Table 2) with reference to macroanatomical brain atlases (Rorden and Brett, 2000). For the six go/no-go blocks, the two raters manually inspected the time-series data and removed the blocks with sudden, obvious, discontinuous noise generated by the motions of the subjects. Cohen's Kappa coefficient for inter-rater consistency was 0.94. The data for 11 subjects were analyzed because we excluded the data for subjects with more than three out of six blocks removed.

**Table 2:** Spatial profiles of target channels.

**CH**	**MNI coordinates** ***x, y, z (SD)***	**Macroanatomy**	**Prob.** ** (%)**	**Brodmann area**	**Prob.** ** (%)**
10	49, 46, 25 (11)	R middle frontal gyrus	54.3	45 pars triangularis Broca's area	67.4
		R inferior frontal gyrus	45.7	46 Dorsolateral prefrontal cortex	32.6

*Prob., probability; SD, standard deviation; R, right*.

We examined the effects of medications between post- and pre-medication contrasts for each medication (GXR or placebo) for the primary analysis. We observed no significant main effects or interactions of a medication type and age in months (two-way mixed ANCOVA, *Fs* < 0.20, all *ps* > 0.05; Table 3).

**Table 3:** fNIRS data for ROI analysis.

**(a) Means and standard deviations**								
	**GXR-to-placebo (*****n*** **=** **4)**				**placebo-to-GXR (*****n*** **=** **7)**			
	**intra-GXR**		**intra-placebo**		**intra-GXR**		**intra-placebo**	
	***M***	***SD***	***M***	***SD***	***M***	***SD***	***M***	***SD***
CH10 Δoxy-Hb (mM·mm)	−0.01	0.05	0.04	0.06	0.05	0.06	0.01	0.08
**(b) Two-way mixed ANCOVA for medication type and medication order**								
**Source**		**df**_**1**_ **, df**_**2**_		***F***		***p***		***ES***
Main effect of medication type (intra-GXR vs. intra-placebo)		1, 8		0.010		0.923		0.001
Main effect of medication order (GXR-to-placebo vs. placebo-to-GXR)		1, 8		0.197		0.669		0.024
Interaction of medication type and age in months		1, 8		0.006		0.938		0.001

*M, mean; SD, standard deviation; df_1_, degrees of freedom 1; df_2_, degrees of freedom 2; F, F-value; p, p-value; ES, effect size*.

### fNIRS Data 2: Exploratory Analysis

We examined the effects of medications between GXR^post−pre^ and placebo^post−pre^ for the exploratory analysis of oxy-Hb signals, using the CBSI method.

**Figure 3:**
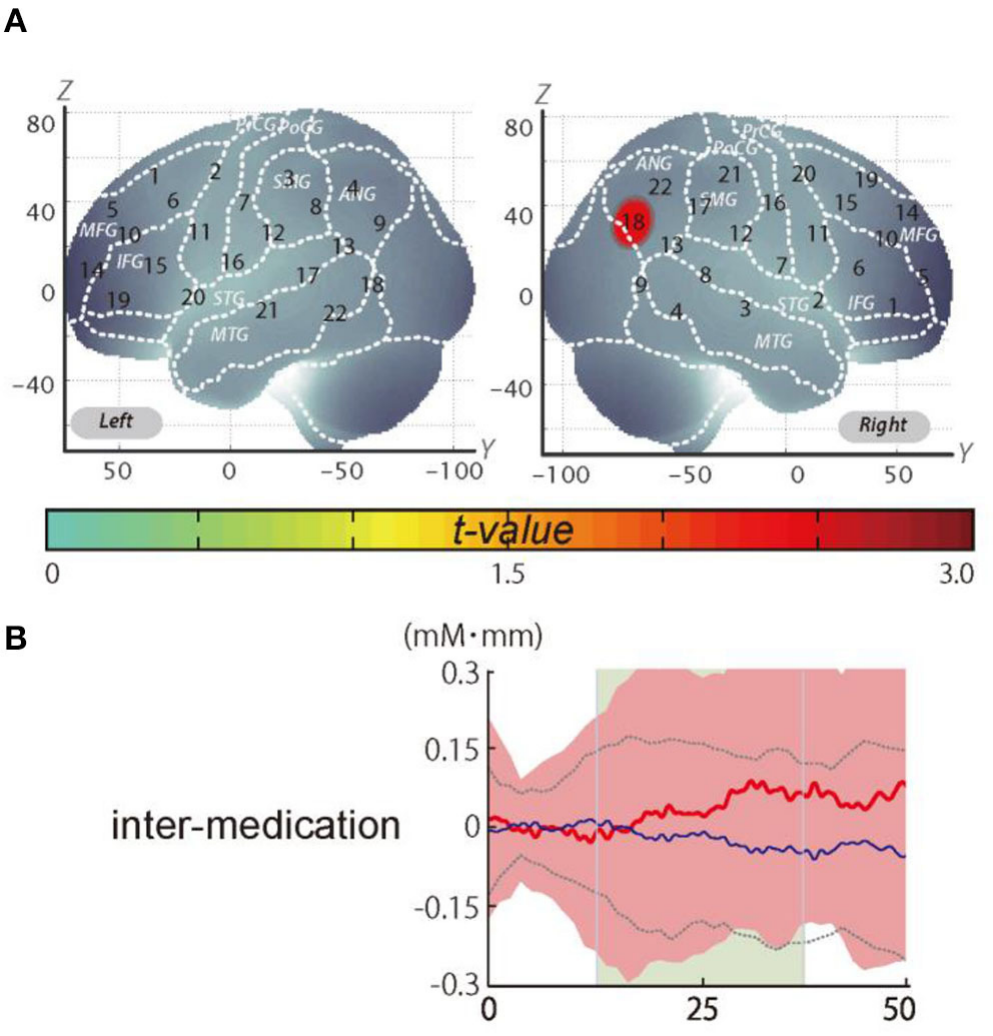
The results of the exploratory analysis. **(A)** Cortical activation patterns during the go/no-go task. The *t*-map for standardized oxy-Hb signals is displayed with significant *t*-values (one sample *t*-test) shown according to the color bar. **(B)** Waveforms of standardized oxy-Hb (red line) and deoxy-Hb (blue line) signals for right CH18. The green area indicates the period of analysis (from 4 to 24 s after the go/no-go block onset).

It should be noted that we did not observe a significant change when applying the M_eff_ method to correct the multiplicity due to the multichannel measurement. However, in data uncorrected for family-wise errors by the M_eff_ method, we observed a significant change in the oxy-Hb signal with a medium effect size at the right CH18 for inter-medication (one sample *t*-test, *p* < 0.05, uncorrected by the M_eff_ method, Cohen's *d* = 0.71; Table 4, Figure 3).

**Table 4:** fNIRS data for exploratory analysis.

	**inter-medication(GXR**^**post-pre**^ **vs. placebo**^**post-pre**^**)**					
	***N***	***M***	***SD***	***t***	***p***	***ES***
CH18 Δoxy-Hb	12	0.047	0.065	2.474	0.031^a^	0.714
(mM·mm)						

*M, mean; SD, standard deviation; t, t-value; p, p-value;*

a *p < 0.05 (uncorrected); ES, effect size*.

We set the right CH18 as an ROI for this analysis; this channel was located in the border region between the right AG and MOG (MNI coordinates *x, y, z* (*SD*): 56, −69, 32 (13); AG, 73%; MOG 27%; Table 5) with reference to macroanatomical brain atlases (Rorden and Brett, 2000).

**Table 5:** Spatial profiles of the channels screened for involvement with go/no-go tasks.

**CH**	**MNI coordinates** ***x, y, z (SD)***	**Macroanatomy**	**Prob.** ** (%)**	**Brodmann area**	**Prob.** ** (%)**
18	56, −69, 32 (13)	R angular gyrus	73.0	39 Angular gyrus, part of Wernicke's area	97.4
		R middle occipital gyrus	27.0	22 Superior temporal gyrus	2.6

*Prob., probability; SD, standard deviation; R, right*.

### Behavioral Data

The results of the second analysis of average accuracy for go and no-go trials and RT for correct go trials in the go/no-go block for the inter-medication contrast (GXR^post−pre^ vs. placebo^post−pre^) comparisons are summarized in Table 6. We found no significant differences in RT for correct trials or in accuracy for the go and no-go trials (Table 6).

**Table 6:** Behavioral data.

	**inter-medication(GXR**^**post-pre**^ **vs. placebo**^**post-pre**^**)**					
	***N***	***M***	***SD***	***t***	***p***	***ES***
RT for correct trials (ms)	12	−9.920	83.852	−0.410	0.690	−0.118
Accuracy for go trials	12	−0.032	0.196	−0.572	0.579	−0.165
Accuracy for no-go trials	12	−0.100	0.238	−1.446	0.176	−0.417

*Performance data (RT for correct trials, the accuracy rate for go and no-go trials) are presented from go/no-go blocks. M, mean; SD, standard deviation; t, t-value; p, p-value; ES, effect size; RT, reaction time*.

## Discussion

### Overview

This clinical study explored the neuropharmacological effect of GXR with a randomized, double-blind, placebo-controlled, crossover study of pediatric ADHD patients. The subjects had been administered 1-mg tablets of GXR continuously for at least 8 weeks before the start of this study, and who showed clinical effects based on the clinical course. In the primary analysis, the results revealed no significant differences in oxy-Hb signal changes in the primary endpoint of efficacy, which was the brain ROI [right prefrontal area: inferior frontal gyrus, middle frontal gyrus (IFG/MFG): right CH10]. On the other hand, in the *post-hoc* analysis, the verification of the exploratory endpoint in brain regions other than those set as ROIs exhibited significant differences in oxy-Hb concentration signal changes in the right CH18 (right angular gyrus). Based on the results of the current study, the right angular gyrus, known as an important component involved in the attention function network (Seghier, 2013), was presumed to be involved in the brain functional pharmacological action of GXR.

### Behavioral Performance for go/no-go Task

In recent years, there have been many reports on brain function studies that visualize brain activation patterns dependent on cognitive function tasks, which clarify the pathophysiology of neurodevelopmental disorders and mental disorders (Chen et al., 2011).

In the go/no-go tasks used in this and other studies, neuroimaging modalities, such as fMRI and fNIRS, were applied during the task, leading to the clarification of the pathophysiology associated with ADHD such as behavioral inhibition, attention inhibition, working memory, and so on (Menon et al., 2001). In addition, the behavior analysis indicators of go/no-go tasks, the correct response rate of go tasks (omission errors), the correct response rate of no-go tasks (commission errors), and the reaction time (abbreviated as RT) of go tasks are independent parameters, and each of them reflects a specific function of ADHD. It has been reported that a decline in the correct response rate of go tasks reflects inattention, and that a decline in the correct response rate of no-go tasks and a delayed RT reflect impulsivity (Barkley, 1997). Some reports indicate that, after OROS-MPH administration, the correct response rate of no-go tasks, the correct response rate of go tasks, and RT improved in both pediatric and adult ADHD patients (Aron et al., 2003).

In this study dealing with the differences between before and after GXR administration and before and after placebo, there were no significant differences in behavior analysis results. This could be due to the small number of subjects and the experimental procedure in which the subjects were administrated GXR after a washout period of 4 days, following at least 8 weeks of daily use. In addition, in our previous fNIRS studies, the inconsistencies in behavioral parameters were observed (Monden et al., 2012b, Nagashima et al., 2014), although significant neural responses to medication in children with ADHD have been consistently shown. Therefore, hemodynamic activation patterns might visualize the mode of inhibition deficits more robustly than do behavioral parameters in ADHD children.

### fNIRS Examination of go/no-go Task and GXR Effects

In the current study, we formed the hypothesis that the activation of the right prefrontal area observed after the oral administration of OROS-MPH or ATX in our previous study (Sutoko et al., 2020) would also be observed after GXR oral administration in the primary analysis. Based on this hypothesis, we specified the area corresponding to the right prefrontal area as the region of interest. However, there were no significant changes in the differences between before and after GXR oral administration and before and after placebo oral administration.

Bédard et al. (2015) performed fMRI measurement during a go/no-go task after 6–8 weeks of continuous oral administration of GXR and placebo on the pediatric ADHD patients (aged 8–15) (Bédard et al., 2015), which is the only reported fMRI study of the ADHD patients. However, their study observed neither activation in the prefrontal area nor in any other brain regions, including the angular gyrus. They postulated that the reasons for this include the possibility of statistical Type II error for noradrenaline reuptake inhibition, the pharmacological action of OROS-MPH and ATX, and the different actions of the α_2A_ receptor agonists of GXR, or to a lack of observation of the effects of a single-dose administration without withdrawal, unlike in the current study.

In other previous reports, there have been different views on the drug efficacy responses of GXR on the prefrontal area. Only two fMRI studies measured the neural responses of healthy young adults with single oral doses of GXR or a placebo in a double-blind, counterbalanced design as in our current study. Schutz et al. used an emotional go/no-go task (Schulz et al., 2013), and Clerkin et al. used a warning cue task (Clerkin et al., 2009), one type of an attention task, and both of them showed response-related activation in the left dorsolateral prefrontal cortex (DLPFC). We have been unable to find any studies focusing on healthy children and adolescents to date.

In addition, in basic animal studies, various actions of GXR have been reported. There are reports stating that α_2A_ receptor agonists, including GXR, reduced noradrenaline (Devoto et al., 2003) and enhanced signal transduction *via* hyperpolarization-activated cyclic nucleotide-gated (HCN) channels in the postsynaptic membrane (Arnsten et al., 2007).

In the current study, however, the *post-hoc* analysis revealed significant activation in the AG. We corrected the oxy-Hb signals of all the channels for motion artifacts, using the CBSI method (Cui et al., 2010). We performed a one-sample *t*-test (two tails) on the inter-medication contrast with an alpha level set at 0.05. The results did not reveal a significant change when applying the M_eff_ method to correct the multiplicity due to the multichannel measurement (Uga et al., 2015). However, we observed a significant change in the oxy-Hb signal in the right CH18 for inter-medication [one sample *t*-test, *t*_(11)_ = 2.47, *p* < 0.05, uncorrected by the M_eff_ method, Cohen's *d* = 0.71, Table 4] without family-wise error correction. The small sample size of the current study might be the cause of the insignificance in the family-wise error correction. However, we evaluated the activation on all the channels based on the effect size. As a result, we confirmed that the activation on CH18 exhibited a sufficiently large effect size (Cohen's *d* = 0.71). The effect size was larger than the effect size of ATX administration seen in a previous study (Nagashima et al., 2014).

The frontoparietal network is known to be involved in attention inhibition; in particular, the attentional flexibility and bottom-up attention function (Peers et al., 2005; Cabeza et al., 2008) are said to show activation of the inferior parietal cortex (including the supramarginal gyrus and the angular gyrus).

In our previous study, when the ADHD group and the typical development group were compared, less activation of the right prefrontal area and the right angular gyrus during the go/no-go task was observed in the ADHD group. On the other hand, the right prefrontal area and the right angular gyrus were significantly activated in the ADHD group after OROS-MPH oral administration. Based on the above, the activation of the right angular gyrus observed after GXR oral administration in this study is thought to reflect neuropharmacological activations of one of the attentional components that exhibit dysfunction in ADHD children.

When considering the results of the pharmacological action of GXR, a variety of reports have stated that it enhanced the signal transduction of noradrenaline *via* HCN channels in the postsynaptic membrane (Arnsten et al., 2007), also suggesting its relationship with the activation of the right angular gyrus after GXR oral administration observed in this study. In that case, it is highly likely that GXR acts on the frontoparietal network, leading to the activation of not only the right angular gyrus but also the right prefrontal area. Nevertheless, there were no significant changes in the results of this study. We speculated that the activation of the right angular gyrus induced by GXR was associated with the bottom-up attention function. Attention function can be mainly categorized into bottom-up and top-down attention. Both systems involve the frontal and parietal cortices network. However, it is thought that the activation patterns of localized areas of the brain differ when each attentional function is activated. Specifically, it has been reported that bottom-up attention activates the parietal lobe first and spreads to the entire frontoparietal network, while top-down attention does the opposite (Buschman and Miller, 2007). Considering the results of our current study, it was speculated that the activation of the parietal region, which was seen as an acute effect of a single-dose GXR administration, visualized the activity of the bottom-up attention function. On the other hand, since frontal and parietal cortices have strong synchrony, long-term administration of GXR may induce both of frontal and parietal cortices activation. Based on the above, further verification of the pharmacological effect of GXR will be necessary in the future.

In this study, the activation of the right prefrontal area was not observed. This could be due to the small number of subjects or to performing brain function measurements 3 h after oral administration despite the Tmax of GXR being 5 h. In the current study, the waiting period to achieve an acute effect of GXR was determined based on previous pharmacological studies, showing that mean values of GXR concentration were over 80% of peak exposure (Cmax) at 3–8 h after a single administration. On the other hand, a sufficient waiting period of 5 h, which is the time to Cmax of GXR, should have been considered. A longer waiting time might affect the slight changes in the right PFC activation.

In our previous study, activation of the right prefrontal area was observed after OROS-MPH oral administration in ADHD-only cases, but activities decreased in cases combined with autism spectrum disorder (ASD) (Sutoko et al., 2019). The neurophysiology of ASD forms a wide spectrum, but, in the previous studies, the pathophysiology of ASD that led to lower activation of the right prefrontal area has not been clarified. In addition, activation of the right angular gyrus has not been shown.

In this study, using DSM-5, pediatric neurologists cautiously diagnosed ADHD and excluded ASD, but the possible influence of comorbid ASD as part of the pathophysiology cannot be excluded. Therefore, in future studies, it is necessary to verify the effects of GXR in cases with comorbid ASD and in cases combined with other mental disorders. In addition, the clinical symptoms and severity of the ADHD of the current participants were not evaluated, using tools such as the ADHD Rating Scale, Clinical Global Impression-Improvement Scale, and DSM-5 categories. This should also be addressed in future studies.

It is also desirable to enhance verification by increasing the number of subjects and keeping the above limitations in mind.

### Special Note

The current research was carried out appropriately based on the research plan. There were no serious adverse events after taking GXR or the placebo, and the study was safely performed. As a result of our careful response to the spread of COVID-19, which was difficult to predict, recruitment of subjects was extremely difficult compared with previous studies, and the target number of cases was not reached. Nevertheless, informed consent of the subjects was obtained, and general measurements were completed without any problems by thoroughly protecting the subjects and the examiners from infection based on the infection-control policies of each medical facility.

## Conclusion

In this specific clinical study, the neuro-functional pharmacological effect of GXR was verified, using the fNIRS measurement method during the execution of a go/no-go task. This study presents the first finding in the world, confirming activation in the right AG, which is thought to reflect a pharmacological functional change in the brain generated by GXR. This effect was verified with a double-blind, randomized, controlled trial on the pediatric ADHD patients. The right AG is one of the attention function networks, and it was considered to have reflected the pharmacological brain function changes of GXR for ADHD. On the other hand, as the activation of the right prefrontal area was not shown, careful verification is necessary for future research.

## Data Availability Statement

The raw data supporting the conclusions of this article will be made available by the authors, without undue reservation.

## Ethics Statement

The studies involving human participants were reviewed and approved by the Ethics Committees of Jichi Medical University Hospital and the International University of Health and Welfare (CRB3180003). The study was registered to the specified clinical trials (clinical trial plan number: jRCTs031190060) as Optical Topography based Neuropharmacological effect of Guanfacine Hydrochloride in ADHD Children. Written informed consent to participate in this study was provided by the participants' legal guardian/next of kin.

## Author Contributions

TY, YM, and ID conceived the presented idea. AI, TH, TT, and YK developed the theory and performed the computation. AI, TH, and SS worked out almost all the technical details. AI, ID, and YM verified the analytical methods. TI, AI, ID, and YM wrote the manuscript, with help from TT. AM and TY supervised the project. YM was responsible for the overall content as a guarantor. All the authors provided critical feedback and helped shape the research and the manuscript.

## Conflict of Interest

This study was funded by Shionogi & Co., Ltd. and Takeda Pharmaceutical Company Limited. The funders were involved in the design of the study and reviewed the manuscript for publication but had no role in conducting the study: collection, management, and analysis. YM reported receiving lecture fees from Nobelpharma Co., Ltd., Eli Lilly Japan K.K., Shionogi & Co., Ltd., and Takeda Pharmaceutical Company Limited. TY reported receiving a research grant from Eisai Co., Ltd. And lecture fees from Novartis Pharma K.K. ID reported receiving a research grant from Saizeriya Co., Ltd., Nichirei Corporation, Kasugai Seika Co., Ltd., and Shiseido Company. Limited. AM was a full-time employee of Hitachi, Ltd. and holds stock in Hitachi, Ltd. and Company. SS is a full-time employee of Hitachi, Ltd. YM, ID, SS, and AM have a licensed patent (WO2017142732, WO2016189955, and US 10,835,169 B2) outside of the submitted work. The remaining authors declare that the research was conducted in the absence of any commercial or financial relationships that could be construed as a potential conflict of interest. The reviewer MY declared a past co-authorship with the authors TI, TT, TY, ID, and YM to the handling Editor.
